# Suicide, neuroinflammation and other physiological alterations

**DOI:** 10.1007/s00406-023-01584-z

**Published:** 2023-03-13

**Authors:** Sabina de la Paz Bengoechea-Fortes, María Jesús Ramírez-Expósito, José Manuel Martínez-Martos

**Affiliations:** Experimental and Clinical Physiopathology Research Group CTS-1039, Department of Health Sciences, School of Health Sciences, University of Jaén, Campus Universitario Las Lagunillas, 23071 Jaén, Spain

**Keywords:** Suicide, Neuroinflammation, Suicide attempt, Inflammatory cytokines

## Abstract

Suicide is considered one of the major public health problems worldwide, being the second leading cause of death in the 15–29 age group. It is estimated that every 40s someone in the world commits suicide. The social taboo surrounding this phenomenon as well as the fact that suicide prevention measures currently fail to avoid deaths from this cause, means that more research is needed to understand its mechanisms. The present narrative review on suicide tries to point out several important aspects, such as risk factors or the dynamics of suicide, as well as the current findings in the field of physiology that could offer advances in the understanding of suicide. Subjective measures of risk such as scales and questionnaires are not effective alone, whereas the objective measures can be addressed from physiology. Thus, an increased neuroinflammation in people who take their own lives has been found, with an increase in inflammatory markers such as interleukin-6 and other cytokines in plasma or cerebrospinal fluid. Also, the hyperactivity of the hypothalamic–pituitary–adrenal axis and a decrease in serotonin or in vitamin D levels seems to also be involved. In conclusion, this review could help to understand which factors can trigger an increased risk of dying by suicide, as well as pointing out those alterations that occur in the body when someone attempt to commit suicide or succeeds in taking their own life. There is a need for more multidisciplinary approaches that address suicide to help to raise awareness of the relevance of this problem that causes the death of thousands of people every year.

## Introduction

Suicide is a multifactorial phenomenon that consists of intentionally and voluntarily taking one's own life through a self-directed harmful act. It is one of the world's leading public health problems and is estimated by WHO to be the cause of one million deaths each year, making it the second leading cause of death in the 15–29 age group [7].

Suicide can be approached from different perspectives that help us to understand it, finding explanations from philosophy, psychology, sociology or physiology. Despite the fact that suicide has been an issue for the scientific community for years, its approach has been hindered by the lack of information and the social rejection it provokes. Shedding light on suicide has become a challenge for society and for science, which tries to reduce, sometimes unsuccessfully, its truly worrying mortality rate.

To speak of a completed suicide would be meaningless if we do not first point to the suicide attempt. Suicide attempt could be defined as potentially harmful behaviour that occurs with the intention to die. The difference between this and completed suicide is that the suicide attempt does not end the person's life [8].

To raise awareness of the problem and give it relevance, 10 September was proposed as World Suicide Prevention Day by the International Association for Suicide Prevention and the World Health Organization. In 2013, the WHO created the first Mental Health Action Plan to reduce suicide cases worldwide, setting the goal of reducing the number of people who commit suicide by 10% by 2020 [48].

## Epidemiology

It is a difficult task to talk about the data that exist on suicide. The lack of clarity surrounding this issue is due, among other things, to the great social impact and the conception of suicide as a taboo subject in our society. It is believed that the actual number of people who die by suicide may be higher than reported. This is why it is necessary to talk about suicide and to investigate it further. However, there are studies that agree on certain aspects of this phenomenon [7].

It is estimated that more than 800,000 people commit suicide every year, i.e. every 40s someone in the world commits suicide. The World Health Organisation states that by 2030 this figure will rise to one million suicide deaths per year [7, 48].

Looking at suicide attempts, we find that for every death by suicide there are 20 or more suicide attempts. This shows that the number of people who attempt suicide per year is around 16 million, a truly worrying fact for global health. This is one of the most reliable predictors of suicide that currently exists, and it is of great help in the proper reporting and prevention of suicide deaths [18, 48].

Other noteworthy parameters would be that 80% of suicide cases occur in low- and middle-income countries [18]. Men have higher suicide rates compared to women, up to three times as many cases, but women make more suicide attempts. In addition, men are more likely to use more violent methods of suicide. However, women are more likely to have suicidal ideation [7, 18]. If we look at the age groups, we find that it is the second leading cause of death in the 15–29 age group, and the leading cause of death in women aged 15–19 [24]. The highest percentages of suicides, if we compare all age groups, would be found in people aged 70 years and older [8, 18]. Ninety per cent of people who commit suicide have a mental disorder and more than 50% of people who commit suicide have major depression or bipolar disorder. Studies suggest that only 5% of mental health patients commit suicide [47]. Major depression increases the risk of suicide by as much as 20 times [42]. In addition, 30% of people with treatment-resistant depression attempt suicide at least once in their lifetime [47]. Certain studies show that more than 40% of people who commit suicide went to the emergency department once or more in the last year before death, and 28% went to the emergency department more than three times. This points to a lack of training and methods to detect cases of people who will eventually take their own lives [7]. Some studies point to the influence of the seasons on the phenomenon of suicide, with indications of an increase in suicide deaths in spring and a decrease in winter. Although more large-scale studies are needed to complete this, it is thought that this may be due to temperature, social activities, changes in serotonin levels or sunlight. The increase in suicides in spring could also be explained by a social pressure for renewal and change with the arrival of good weather and the failure to achieve real renewal. Other studies point to notable differences between countries on this issue, making it difficult to know how much influence the seasons have [50]. Looking at the days of the week, we find that there is an increase in suicide cases on Mondays and a decrease on weekends. This could be explained by an increase in social interaction and a decrease in stressors on weekends, and a failure of renewal on Mondays. Just as there is a perception of a new beginning with spring, so it could be with Mondays and the beginning of a new week. However, more studies are needed to be able to say for sure [39].

## Phases of suicidal behaviour

### The four-phase model

Suicide also has its own dynamics, which can be described in four phases as follows. In the first phase, suicidal ideation takes place, which may be expressed verbally or non-verbally. The second phase is the planning of the suicide, a stage of ambivalence that leads to the strategy to be used for the suicide. Once the decision has been made, this is the third phase or the suicide attempt, which fails. Thus, the last phase would be suicide, where the person would finally end his or her own life [5, 37].

### The three-phase model

These four phases are not the only ones that have been proposed to try to explain this phenomenon, and thus the three-step theory of suicide has emerged [23]. In this theory, the concepts of grief, hopelessness, suicidality and connectedness are important.

Firstly, there is the desire to commit suicide. Studies suggest that this arises from a combination of hopelessness and feelings of intense pain. However, if a person is suffering emotionally but has hope for an improvement in the future, he or she will try to overcome the situation and show an effort to try to remain committed to life.

Secondly, there is an intensification of suicidal desire, which is mostly moderate. While pain takes away the feeling of wanting to live, there is also a connection that binds the person to wanting to go on living. If a person has a higher level of pain compared to their level of connection to life, their desire for suicide will be increased.

The last step of this phenomenon is the occurrence of suicide attempts. At this stage, people have an intense desire to commit suicide.

However, not all those who are suicidal in their approach finally commit suicide. Experts point to fear of death as one of the main factors influencing not ending life by suicide. A possible ability to cope with fear and pain through previous experiences such as sexual abuse, the health profession or having experienced non-suicidal self-harm could reinforce this idea. This three-step theory would also be supported by the influence of factors such as personality, genetics of the individual or access to knowledge about methods of suicide [23–25].

## Methods of suicide

One of the aspects to be taken into account in the act of suicide is the method used, which can be classified into violent methods (firearms, hanging, precipitation, among others) and non-violent methods (poisoning or overdose with drugs or medicines) [8]. The lethality of a method is determined by the time between the use of the method and death, and the availability of medical help. Both factors are interrelated at the same time, as the more lethal methods decrease the likelihood that medical services can be called upon to prevent death [51].

According to the WHO, the methods most commonly used among the suicidal population are hanging, poisoning and firearms, the former being the most common due to its wide availability. Differences can also be found between men and women, with men using more lethal methods than women. After hanging, the most common methods used by men are firearms and, in third place, poisoning. In contrast, for women, drug poisoning and, in third place, precipitation from a building are the most frequently used methods [51].

## Risk factors

**Fig. 1:**
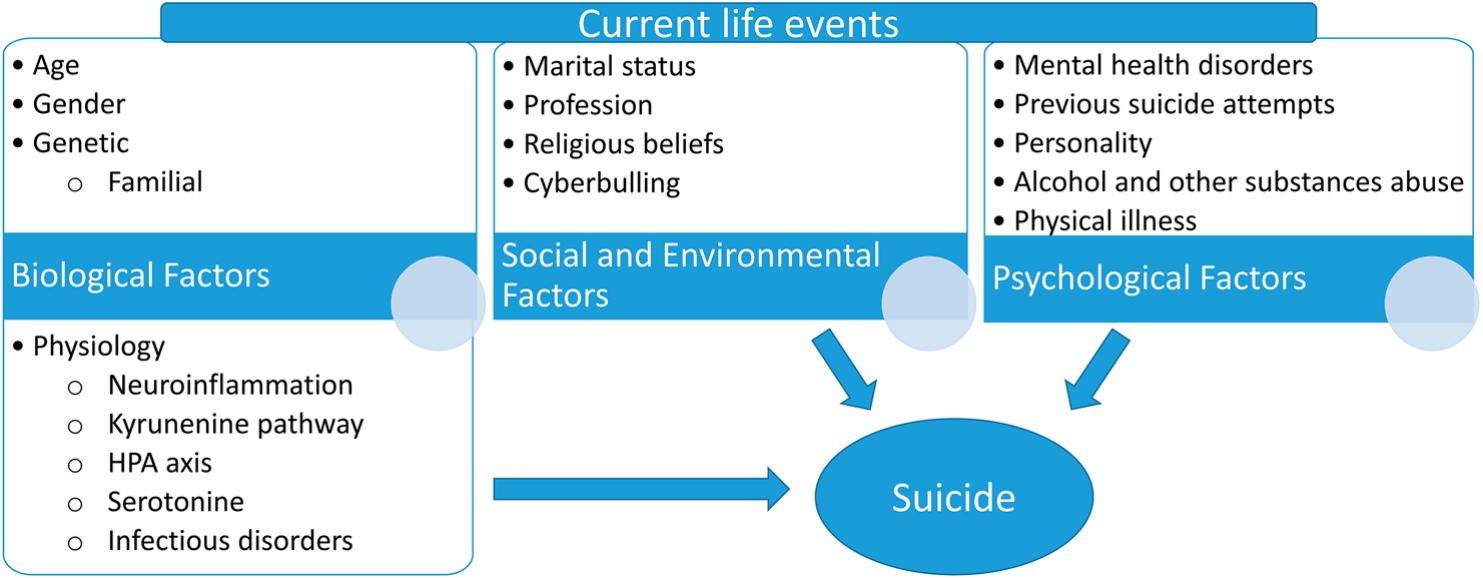
Drivers of suicide risk

To talk about suicide is also to talk about its risk factors, as they have a significant influence on suicidal behaviour. These can be classified into biological (including familial), mental and physical health (psychological) and social (including environmental) factors (Fig. 1).

Age is one of the risk factors to be highlighted, as it represents the second leading cause of death in the 15–29 age group. We also found that the age group with the highest rate of people who take their own lives is the over-70s, with a higher rate in the over-75s [20]. According to the WHO, suicides increase with age. This may be partly explained by a decrease or lack of interpersonal relationships and a decrease in daily activity. In terms of suicide attempts, suicide attempts are more frequent in women aged 15–24, while in men, they are more frequent in the 25–34 age group [20].

Regarding gender, we found that women have more suicidal ideation and attempts, 3–4 times more than men. However, more men commit suicide, 2–4 times more [20]. In both men and women, the risk of committing suicide is substantially increased in schizophrenia, substance abuse and affective disorders, with schizophrenia being of particular relevance in men and substance abuse in women [20]. In addition, some events that are considered protective factors for both sexes are pregnancy and having a son or daughter [5].

For genetic factors, an association has been found between genetic polymorphisms and suicidal behaviour. In addition, it has been found that the odds of suicide are ten times higher in those who have had family members who committed suicide. It is also estimated that approximately 43% of suicidal behaviour can be explained by genetics [5]. Reviewing family history is important, as those children who have had parents who have committed suicide are up to four times more likely to take their own lives. Some studies point to the age of the child when a parent commits suicide, noting that the younger the age of the child, the greater the likelihood of suicide in the future. The reason for this is currently unknown. It could be a genetic issue or parental imitation [18]. In addition, reviewing family history may also involve parental neglect or physical or sexual abuse, which may increase the risk of children taking their own lives [7]. It is believed that those who suffered sexual or physical abuse in childhood may be the cause of suicide in 50% of cases in females and 30% in males [8], which should also be taken into account.

Studies linking suicidal behaviour and molecular genetics are now trying to find specific genes that explain it. Some point to specific loci in chromosomes that play an important role in suicide. Others point to the importance of mental disorders and their heritability, which would link the two phenomena and could thus be explained. Others argue that behavioural factors such as impulsivity or aggressiveness could play a role in genetic factors [16]. However, the studies conducted so far use small population samples and, for the most part, do not differentiate whether people have psychiatric disorders or not, which makes it difficult to provide a concrete explanation of the relationship between suicidal behaviour and genetics [16].

Regarding mental health disorders, if we look at the percentages linking a mental disorder and the phenomenon of suicide, we find that 90% of people who commit suicide have a mental disorder [24]. It is estimated that in higher income countries, the most frequent mental disorders are major depression, bipolar disorder and post-traumatic stress disorder. However, in developing countries, substance abuse disorder and post-traumatic stress disorder are found to be the most prevalent [24].

Some remarkable data are that people with major depression have up to 20 times more risk of committing a suicidal act and up to 80% more risk of suicidal ideation than those who do not have it. If we look at individuals with bipolar disorder, we find that 20–60% attempt suicide. And, in the case of people with schizophrenia, the risk of suicide is 30–40 times higher than in healthy individuals, and 25–50% will attempt suicide. Other studies, however, suggest that people with schizophrenia have an 8.5 times higher risk of suicide [5]. Looking at the average age of people with schizophrenia who commit suicide, we find that they are on average 33 years old [20]. In addition, it has been found that approximately 20% of people who are socially distressed will attempt to take their own life at least once [5].

Other findings point to a close link between eating disorders and suicide, with an eight-fold increased risk of suicide appearing in anorexia nervosa and up to five-fold in bulimia nervosa. There is also a link between attention deficit hyperactivity disorder (ADHD) in men aged 5–24 years, showing a three-fold increase in the likelihood of suicide [20, 29].

Another disorder that could be related to suicidal behaviour is borderline personality disorder (BPD), finding that 10% of people with this disorder will end up committing suicide. Some studies claim that a person with this disorder will have up to three suicide attempts in their lifetime, in most cases due to substance intoxication. A characteristic feature of BPD is chronic suicidal ideation, with increasing intensity of stressful events, which becomes frequent on a daily basis [36]. One of the issues being investigated in relation to this risk factor is the psychotic experience which, although it can be defined as an experience full of hallucinations and delusional moments, is believed to be close to the suicidal act, finding a three-fold increase in the probability of attempting suicide. However, psychotic experiences may be a reflection of suicidal thoughts that already exist in the person and not the cause of it. So far, there is no scientific consensus that clearly supports this relationship [49].

Previous suicide attempts are the most important risk factor for suicide. Some findings that show this close relationship are that 50% of those who committed suicide had made previous suicide attempts or that a suicide attempt increases the risk of attempting suicide again by 32%. Some studies point to a 100 times higher risk of suicide in those who make a suicide attempt compared to those who do not [20].

Regarding personality, two of the most prominent personality traits are hopelessness and impulsivity. The former appears in up to 90% of those who commit suicide [20]. Regarding impulsivity, there are studies that strongly support that this trait influences the transition from suicidal thinking to action, while other studies cast doubt on this [24]. Other related traits are emotional instability (which may increase the risk by 2.3 times), perfectionism and high demands [20].

Alcohol and other substances use/abuse are implicated in 25–50% of suicidal acts and 70% of suicides in adolescents [9, 47]. If we look at the different substances that are related to suicide, we find that those who consume alcohol are 8.5 times more likely to commit suicide [20], which may be involved in an increase in aggressiveness and impulsivity, as well as influencing the use of more radical methods to take their own lives [47]. In recent years, both phenomena have been increasing simultaneously. If we delve deeper into this relationship, we find that acute alcohol intoxication and chronic alcohol consumption increase the likelihood of suicidal behaviour and suicidal ideation. The day after substance abuse, the probability of dying from a suicidal act increases sevenfold [41]. A link has also been found between alcohol intoxication and the use of more lethal methods of suicide, which would increase the likelihood of death. In addition, more than 30% of people who die by suicide had consumed alcohol, 63% of whom were intoxicated [41].

Tobacco has also been associated with suicide and increased impulsivity and aggressiveness, as have marijuana, cocaine and amphetamines. Other substances such as heroin (14 times more likely to be suicidal) or MDMA (9 times more likely than non-users) have also been linked [20]. Finally, studies suggest that LSD, due to its effects such as well-being, optimism and mood enhancement, may be excluded from the other risk factor substances, although this is not a clear-cut statement [9].

Regarding physical illness, several studies suggest that it was present in 25% of cases of suicide and in 80% of cases if the person is elderly. It is believed that there is an increased risk of suicide within six months of being diagnosed with the illness. In addition, it is essential to consider pain as a companion to physical or disabling illness.

Cases are more frequent in patients with HIV and multiple sclerosis. In the latter, mortality rates are twice as high and are more frequent in young men [20].

Several social factors are also included as risk factors. A notable risk factor is a person's marital status, with up to three times the risk of suicide found in divorced or widowed men. Living alone or lacking a social support structure may increase the risk. Some studies point to marriage as a protective factor against suicide [5].

An increased risk of suicide in both unemployment and in some professions has been found. If we look at unemployment, we find that there is a two to three-fold increase in the likelihood of suicide, as well as in those who do not have stability in this area such as the 25–34 age group. In addition, some professions have a higher risk of taking their own lives, such as farmers, police or health workers, as they have access to lethal means [18]. If we focus on health care workers, we find that they are considered to be a group at high risk of taking their own lives, especially women. Factors that may play a role include workload, working hours and schedules, anxiety, communication of bad news, stress during the working day and access to lethal means. Some specialties also have an increased risk of suicide such as anaesthesia, psychiatry, general medicine or surgery [14].

In addition, some studies mention religious beliefs as a protective factor for suicide [5].

Finally, suicide is also affected by the twenty-first century and the use of technology in all areas. Cyberbullying in particular has become a risk factor for suicide, with studies indicating that approximately 20% of adolescents are at risk of taking their own lives, and that 78% of those who have done so have been cyberbullied [5].

### The influence of COVID-19 on suicide

A recent historical event such as the COVID-19 pandemic has set the path for new studies that bring a new field of vision in science. It had repercussions in the field of mental health, as the authorities ordered the isolation and the confinement of the population. This, together with the fear of contagion and daily deaths, caused a turning point in scientific literature and in the lives of millions of people around the world. This is why it affected all areas of life, from the psychological to the physical to the economic, among many others. In this section, we will look at the mental health consequences of COVID-19, the groups at risk, its relationship with suicide and the scientific evidence that exists today.

According to studies in the United States, about 40% of adults experienced anxiety or depression in the first months of the COVID-19 pandemic [1]. Considering the repercussions that this pandemic has had on the mental health of the world's population, it has been found that the most frequent symptoms have been stress, anxiety, depression and insomnia. This can be explained by the impact of this virus on all areas of daily life, from home confinement to fear of infection or deaths that occurred when transmission was unknown. This led to a state of social unrest that forced many to be alone, which was a determining factor in the appearance of some symptoms such as anxiety or the risk of suicide. In addition, there was an increase in self-harm [17]. There was also an increase in the number of patients admitted for an intensification of psychiatric symptoms, anxiety and obsessive–compulsive disorders, while at the same time increasing the risk of dying by suicide [3, 12].

It is of particular relevance to note that the impact of COVID-19 on people's mental health occurred both in people who were infected by this virus and those who were not. Because this pandemic affects so many areas, symptoms and complications in the field of mental health can develop in any person. This makes the magnitude of the problem very important and requires global action.

The risk factors for suicide that emerged during the pandemic include: (1) loneliness and isolation. This was due to the isolation required by the authorities, quarantines and mobility restrictions [3]; (2) fear of contagion. This is because in the first few months there was a lack of knowledge about the transmission of the virus, how it affected people and what to do to stop it. Due also to social media, sometimes false or unscientific news appeared, which increased the lack of knowledge and anxiety. As it was an unknown virus, there were no vaccines or treatments that were effective. It was not until months later that health workers were able to decide on one type of treatment or another. In addition, the development of several vaccines against the virus led to a significant drop in the number of people infected [3]; (3) prejudice. Those who were infected by this virus were socially stigmatised, which is a risk factor for mental health [3]; (4) intensified mental health disorders. As noted above, anxiety, stress, depression, substance abuse disorders, among others, appear to have intensified. In addition, due to the overload of work for health personnel, the authorities urged citizens to avoid going to hospitals or primary care centres in order to avoid the collapse of the health system. This meant that many pathologies could not be properly treated or diagnosed, and some intensified their symptoms [3]; (5) employment and economic changes. The isolation of the population and the halt in employment caused many people to lose their jobs or to stop earning an income. Unemployment is believed to have caused an increase in the number of suicides. There were also sectors that forced their workers to work from home, via the internet. Most countries experienced a decline in economic growth, which led to the symptoms mentioned above [3]; (6) increased access to lethal means. Some studies point to the decrease in vigilance due to the pandemic situation to explain the increase in access to metal means of suicide [3].

Also, those groups that were at risk during the first months of the COVID-19 pandemic include: (1) people who were on the front line. The health professionals are particularly important, as they were and are the ones in charge of diagnosing the infection, monitoring and treating the symptoms of the patients, as well as providing support to their families. Working conditions were affected by the situation and health workers had to face a lack of material, longer working hours, fear of contagion, stress, anxiety, loneliness or voluntary isolation in their homes to avoid infecting their relatives. This led to an increase in hopelessness, one of the key aspects in identifying suicide risk [3]. One of the indicators among healthcare workers that indicated an increased risk of developing suicidal ideation was burnout. The healthcare category showing the highest rates of burnout during the pandemic was nursing staff, according to the scientific literature [1]; (2) people in old age. Older age has been associated with both the risk of suicide and the risk of having complications from COVID-19. In this group of people, loneliness, isolation and depression play important roles in the development of suicide risk. A large percentage of them require help with daily activities and, due to home confinement, could not be properly cared for, increasing frailty in this age group [3]; (3) people in adolescence This age group also presents various suicide risks. It should be noted that technology and the social environment play a very important role for adolescents. During the pandemic, there was a disruption of the social environment and an isolation which made increase symptoms affecting mental health. The development of an individual's identity is largely created in this age range, so that in many cases, there was an imbalance due to home confinement. This led to an increased risk of suicide [3]; (4) victims of domestic violence. Because of house confinement, many families were forced to live together for months at a time without being able to leave their homes. This led to an increased risk of violence in families, as in the case of gender-based violence. This group is among the most vulnerable to confinement, as it increases the risk of death by suicide. Psychological support units were also restricted, making victims' coping with these situations more complex [3, 10]; (5) homelessness. This group is at high risk of increased economic, psychological, social and other impacts. An increased risk of suicide has also been reported in this group due to COVID-19.

Despite the need for more scientific evidence on the relationship between suicide and COVID-19, it can begin to deduce that there has been an increase in suicide risk. Sher [43] found that the mental health consequences of the COVID-19 crisis including suicidal behaviour are likely to be present for a long time and peak later than the actual pandemic. Other authors found that despite the initial alarming predictions for an increase in suicide rates due to the COVID-19 pandemic, the majority of published studies to date suggest that experienced difficulties and distress do not inevitably translate into an increased number of suicide-related deaths, at least not in the short-term [15]. A recent meta-analysis found that increased event rates for suicide ideation (10.81%), suicide attempts (4.68%), and self-harm (9.63%) during the COVID-19 pandemic when considered against event rates from pre-pandemic studies [13]. On the other hand, it has been found that by increasing the risk of isolation, fear, stigma, abuse and economic fallout, COVID-19 has led to increase in risk of psychiatric disorders, chronic trauma and stress, which eventually increase suicidality and suicidal behaviour [3]. These findings suggest that the COVID-19 pandemic may lead to an increase in suicide rates.

However, more studies and reviews are needed to provide a more scientifically rigorous coverage of this phenomenon. The lack of cause-of-death records during the pandemic makes it difficult to draw conclusions and study this relationship.

### Suicide risk assessment

Just as it is important to understand the phenomenon of suicide, it is also important to assess a person's risk of committing suicide. This would make it possible to predict, or at least estimate, a person's likelihood of taking his or her own life.

In order to approach assessment, it is necessary to know that there are more subjective strategies such as questionnaires or scales, and more objective ones such as biomarkers, which will be discussed later.

Firstly, objective strategies include the collection of data on the person, the analysis of the family history, the pathologies they present, among others. In short, the assessment of the individual's risk factors through interviews carried out by the professional [21, 48]. Tools have been created to facilitate the access of professionals to the identification of the risk of suicidal behaviour, such as scales or questionnaires. These tools have been approved by the European Psychiatric Association, although the scientific evidence is not high [18]. Some of them are the Beck Hopelessness Scale, the Suicidal Ideation Scale or the Columbia Suicide Severity Rating Scale [18, 47]. These scales focus on identifying the person's suicidal ideation and the risk factors that may accompany it. However, the cooperation of the person is needed to know the reliable results of these tools. This is why previous suicide attempts become one of the most important factors in this phenomenon, as they make it easier to assess suicide risk [47].

Secondly, objective strategies such as biomarkers of inflammation are gaining importance in the scientific community and in methods of suicide assessment and prediction [30, 45]. We will focus on them below and analyse the mechanisms of neuroinflammation that accompany suicidal behaviour. Over the years, more and more scientists have been supporting research into biomarkers of inflammation, as they could be used to reveal to healthcare professionals which individuals are at higher risk of suicide or even who are more likely to commit suicide. Other physiological alterations, which will be discussed below, would also shed light on the mechanisms of suicide. If the subjective strategies discussed above fail to fully unravel this phenomenon, will inflammatory biomarkers allow us to tailor prevention and treatments to combat death by suicide?

### Physiological alterations in suicide

The path that leads to suicide is accompanied by physiological alterations in the body. One of the alterations that stands out from the rest is neuroinflammation. This phenomenon has a large repertoire of scientific literature, so we will focus specifically on inflammatory cytokines and their direct relationship with suicide, and on the kynurenine pathway. Other striking changes reported in numerous studies and to which we will give space in this review are the hyperactivity of the hypothalamic–pituitary–adrenal (HPA) axis, changes in serotonin levels, the relationship between suicide and immune and infectious disorders, and the relationship between suicide and other notable alterations. Although there are several areas that could be addressed in this phenomenon, we will focus on the above-mentioned alterations, as they could lead to major scientific breakthroughs. Some of these disorders may also appear in major depression, as it shares certain characteristics and mechanisms with suicide [8, 34, 46].

Some changes that occur in people who make suicide attempts take place in specific regions such as the hypothalamus, the cortex and prefrontal areas or the hippocampus. Changes in cognition, emotions and decision-making occur in these areas. The anterior cingulate cortex, where the creation of negative self-conceptions is located, is also of importance [47].

#### Neuroinflammation

In order to talk about biomarkers of inflammation and their relationship with suicide, we must first talk about neuroinflammation, a concept that has been studied extensively in recent years. Neuroinflammation is defined as an inflammatory response mechanism of the central nervous system to an alteration produced inside or outside the system, such as trauma, neoplasms, infections, ischaemia or alterations in the immune system, among others. Neuroinflammation is considered a normal mechanism of the brain that contributes to the proper functioning of its structures as long as it is transitory, thus creating a neuroprotective effect. If neuroinflammation spreads over time, creating chronic inflammatory disorders, it could lead to significant damage to the system [27].

The discovery of new findings in this field has brought with it the knowledge that the central nervous system is not isolated. Years ago, the scientific community spoke of the brain as a privileged organ that was isolated by the blood–brain barrier. However, numerous studies have shown that it is in constant communication with the immune system. This is also made possible by the involvement of inflammatory mediators (which can be pro-inflammatory or anti-inflammatory), neurotransmitters or hormones. It is important to know that when inflammation occurs in the central nervous system, glial cells are activated and inflammatory cytokines are released. This is related to the various dysfunctions in the system [27, 32].

Taking a brief look at glial cells, some of them are of particular interest in the field of neuroinflammation. They carry out neuronal support functions. This is the case with microglia, which are a fundamental pillar of central nervous system immunity, bearing certain similarities to macrophages. They go to the sites where inflammation is occurring and, therefore, play an important role in this phenomenon. Astrocytes also contribute to the proper functioning of the immune system, as well as providing neurons with metabolic support and controlling the permeability of the blood–brain barrier. Oligodendrocytes are also involved in neuroinflammation and have receptors for various interleukins. Several studies point to a close communication between microglia and oligodendrocytes, which would offer a more dynamic explanation for inflammation in the nervous system, but further studies are needed to confirm this [27, 32].

As mentioned above, microglia and astrocytes produce inflammatory cytokines. These are messenger proteins that are released in order to regulate immune and inflammatory responses, carry out intercellular communication, as well as participate in cognitive, emotional and behavioural domains that are regulated in the hypothalamus, hippocampus and prefrontal cortex [6, 47]. These particular areas have been found to have a higher number of cytokine receptors [19]. They may be pro-inflammatory, such as IL-1β, IL-6, tumour necrosis factor (TNF-α) or anti-inflammatory, such as IL-10 and IL-4 [19, 32, 42].

##### Neuroinflammation and suicide

In the late 1980s and early 1990s, interferons were being used for the treatment of cancer, hepatitis B and hepatitis C. Soon, health care workers working with these patients discovered a link between interferon treatment and increased depression and suicidal behaviour. The percentage of patients who had depression and were treated with interferon-α in those studies was as high as 30%. This fact attracted the attention of the scientific community, who decided to continue research in this area and discover the relationship between both of them [6, 22, 32, 48].

In this way, inflammation and suicidal behaviour were explored in greater depth, with special interest in inflammatory cytokines. Several studies chose to analyse the brains and various parameters of people who died by suicide. These post-mortem studies found increased inflammation in the brain and cerebrospinal fluid, as well as increased levels of inflammatory cytokines [7]. It has also been shown that those with the highest levels of inflammatory biomarkers are up to three times more likely to commit suicide. This suggests that neuroinflammation and suicide attempts are probably also related [19]. It has also been shown that inflammatory cytokines that exist at the peripheral level can reach the central nervous system through the blood–brain barrier. For this to occur, there must be an increase in the permeability of the barrier, and biomarkers would pass through there. Some studies suggest that those who commit suicide show an increase in the permeability of this barrier and, therefore, an increase in the trafficking of inflammatory cytokines [7]. The most significant alterations that have been found so far with respect to neuroinflammation are (1) increase in interleukin IL-6. IL-6 is the inflammatory cytokine most closely associated with suicide. It can be found increased in plasma, cerebrospinal fluid, as well as in tissues that are analysed when a person has committed suicide. In addition to being related to suicide, it is also related to depression, especially when it is intensified [35, 48]. Increased plasma IL-6 levels raise questions about the occurrence of systemic inflammation [19]. One of the characteristics of IL-6 is that it activates microglial cells [27]; (2) increased interleukins IL-1β, IL-2, IL-4, IL-10 and IL-13. Increased IL-1β occurs in some cortical regions [48]. It is produced by microglial cells, macrophages, lymphocytes, astrocytes, among others. Binding to its specific receptors activates the formation of other biomarkers such as IL-6 and TNF-α. It has been found that in people with multiple sclerosis, this interleukin has higher levels [27]. Regarding IL-2, it has been found to be increased at the same level that its receptors [7], although other authors claim that it is decreased [42]. Altered IL-4 levels can lead to various changes in a person's motivation, emotions and behaviour. In post-mortem studies, increased levels can be localised in the prefrontal cortex of those who die by suicide. However, other studies claim that IL-4 levels are found to be decreased in those with suicidal ideation and suicide attempts. More literature on this interleukin is needed to assess its importance in relation to suicide [19]. Also, altered IL-10 levels, as with IL-4, can lead to changes in motivation, behaviour and emotions [19]. (3) Decrease in interleukin IL-8. Reduced levels of IL-8 have been found in cerebrospinal fluid studies of those who committed suicide. This interleukin has also been linked to anxiety [7]. Also, this interleukin has been studied in relation to hereditary influence and has been found to be related to suicide and genetic factors. In women in particular, this may be more pronounced [26]. (4) Increase in tumour necrosis factor (TNF-α). Several studies have shown an increase in TNF-α in the plasma of those who died by suicide, as well as in the brains of teenagers who also died by suicide [19]. Increased levels of TNF-α appear in people who have attempted suicide. However, it does not occur in people with suicidal ideation [40]. This tumour necrosis factor performs its functions in terms of memory, neuronal plasticity, learning, as well as during sleep, provided when normal levels are present. If, on the other hand, high levels are present, its functions are focused on neuroinflammation and neurodegenerative pathologies [27]. This parameter is also found to be increased in cases of schizophrenia. However, some studies do not consider that there is an alteration, and more studies are needed to be able to state this with certainty [35]. As IL-8, this tumour necrosis factor is influenced by genetic factors [26]. (5) Increase in C-reactive protein (CRP). CRP is a marker used in acute inflammation that is associated with suicidal ideation and behaviour [22]. Those who were more inflamed in terms of CRP, i.e. CRP > 3 mg/L were up to four times more likely to commit suicide compared to those who were less inflamed, which in terms of CRP would be CRP < 1 mg/L [33, 48]. Also, an increase in CRP levels is often accompanied by an increase in the number of lymphocytes [22]. As with TNF-α, increased levels of CRP have been found in those with a suicide attempt, but not in those with suicidal ideation [40]. Although numerous studies support these physiological alterations, others deny the link between suicide and certain parameters. This is the case, for example, with IL-6, IL-4, IL-2 or TNF-α [19].

In addition to suicide, some of these changes in inflammatory cytokines have also been seen in major depression and its symptom intensity. In order to differentiate the relationship between inflammatory biomarkers and suicide, and inflammatory biomarkers and major depression, it would be necessary to conduct studies with large samples in which it would be determined who has major depression and commits suicide, and who commits suicide without having major depression. By making this differentiation in the study sample, it would be possible to know more precisely which biomarkers most influence each of these phenomena [19].

Other topics related to neuroinflammation have been also evaluated. Thus, increased microgliosis, indicating increased inflammation, has been found in people with schizophrenia or major depression who die by suicide [35]. In addition, increased vascular density has been found in the prefrontal cortex of those who committed suicide, specifically in the white matter [7]. An increase in granulocytes has been also found. Increased MAO-B and apolipoprotein-E have also been reported in cases of suicide [42].

Other aspects that must also be considered are the relationship between suicidal behaviour and certain aspects surrounding suicide. Although this field is still to be defined, some studies shed light on some parameters such as (1) aggressiveness and impulsivity. Two aspects of personality that have been found to be linked to various physiological alterations in neuroinflammation are impulsivity and aggressiveness. In both, an increase in IL-6, TNF-α and CRP has been found [7]. (2) Recent suicide attempts. Several studies point to a relationship between the short time interval between the suicide attempt and the increase in the level of inflammatory cytokines. Thus, the more recent the suicide attempt, the higher the level of inflammatory cytokines in the person [19]. (3) Methods of suicide. According to some studies, the method used in suicide also involves certain alterations in physiology. An increase in interleukin-6 appears the more aggressive the method of suicide [31]. (4) Low level of attention. Low attention span has been linked to increased levels of IL-6, as this interleukin carries out some of its functions in cognitive areas of the central nervous system [19]. (5) Child physical abuse. People who have been physically abused as children or adolescents have higher levels of IL-6 and IL-4 [19]. In addition, it has been shown that there is hyperactivity of the HPA axis, a system related to stress, which will be discussed later [18]. (2) Depression. Many studies link suicide to major depressive disorder due to certain common elements, including neuroinflammation. In order to talk about both, it is necessary to know that the intensity of the depression will influence the risk of carrying out the suicidal act [2].

Some research is focused on finding out whether treatments using antidepressants decrease markers of inflammation. This could be of great help in decreasing neuroinflammation in these people and thus decreasing the risk of suicide [35].

Regarding NSAIDs, there are certainly studies that support a possible decrease in the levels of inflammation in those individuals treated with NSAIDs. There is a notable decrease in suicidal ideation in those treated with ibuprofen, celecoxib or naproxen [47]. At the same time, it is suggested that these NSAIDs also pose a risk to those who attempt suicide by taking them. It is believed that up to 50% of people who attempt suicide do so by consuming high proportions of NSAIDs [47].

Regarding alcohol and other drugs, both are related not only to suicide, but also to neuroinflammation. The influence of alcohol on increased levels of IL-6 and CRP is notable. Cannabis, however, is thought to reduce IL-1β levels. Tobacco use, on the other hand, has been linked to increased levels of TNF-α [9].

The link between neuroinflammation and COVID-19 is important to note, as it may also shed light on the link between suicide and COVID-19 described above. Some studies indicate that those infected with this virus show alterations in the central nervous system, leading to neuroinflammation and an increase in inflammatory cytokines. These pathways are also involved in suicide, which is an important aspect to take into account in the field of mental health. In particular, there is an increase in IL-1, IL-2, IL-6, TNFα and CRP. IL-6 is closely implicated in the phenomenon of suicide, as well as IL-2, which is associated with the virus and an increased risk of dying by suicide [10].

Some studies point to those that block the production of inflammatory cytokines, such as tocilizumab or ketamine, as a possible effective treatment for severe symptoms of the virus. Although the scientific community is rapidly trying to expand the field of knowledge about COVID-19, more studies are needed to be able to say for sure [3].

#### The kynurenine pathway

The kynurenine pathway is closely related to inflammation and, in turn, to suicidal behaviour. The kynurenine pathway is the degradation process of tryptophan. It is present in numerous tissues such as brain, liver, gut and immune cells. We know that kynurenine is broken down into quinolinic acid and kynurenic acid, both of which are neuroactive [7, 47]. Several studies point to a link between quinolinic acid and neuroinflammation, showing that some inflammatory cytokines such as IL-1β and IL-6 activate the kynurenine pathway [38, 47]. There is also an increase in quinolinic acid in patients who attempted suicide, including those who attempt suicide violently, being two to three times higher [7]. Those people who have depression and suicidal ideation have higher levels of quinolinic acid than those who have depression and no suicidal ideation [47]. Quinolinic acid is elevated for up to 2 years in those who attempt suicide [7]. Finally, there are elevated levels of quinolinic acid in specific areas of the brain, which may indicate that some regions are more sensitive to neuroinflammation [8], and some treatments such as electroconvulsive therapy or ketamine can disrupt the kynurenine pathway [47].

#### The hypothalamus–pituitary–adrenal (HPA) axis

Another of the most notable alterations in terms of suicide at the physiological level is the hyperactivity of the hypothalamus–pituitary–adrenal (HPA) axis. This is a system that, thanks to its negative feedback mechanism, acts by controlling the levels of cortisol that the body needs. It increases its production if low levels are detected, and stops its production if levels reach the target. The importance of this system lies in the relationship this axis has with stress, as it influences the occurrence of suicidal behaviour [4].

But how does the HPA axis work? Negative feedback triggers the recognition of cortisol levels in the body via mineralocorticoid and glucocorticoid receptors. The hypothalamus then releases corticotrophin-releasing hormone or CRH, which triggers the release of adrenocorticotropic hormone in the pituitary gland or ACTH. This in turn triggers the release of glucocorticoids in the adrenal glands, including cortisol. When the correct levels of cortisol are detected, the release of CRH and ACTH is inhibited, and cortisol production ceases. When these levels fall, the mechanism is triggered again by stimulating the release of CRH and ACTH, and more cortisol is created [4, 8].

However, hyperactivity of the HPA axis may lead to tissue damage and may also cause alterations in stress management and cognitive impairment [8, 47]. Some studies indicate that hyperactivity of the axis may result in cortical hypertrophy, as well as elevated cortisol and CRH levels [8, 47]. However, other studies claim that cortisol levels are lower in people who have attempted suicide [4].

Reference to the HPA axis is essential to understanding suicide. It is suggested that disruption of this axis is also related to mental health disorders, in addition to suicide directly. Hyperactivity of the HPA axis is considered to be a good predictor of suicidal behaviour [4]. This relationship is estimated to increase the risk of suicide by more than 4.5-fold. It is also unknown whether the dysregulation arises from a suicide attempt or whether, on the contrary, it is the hyperactivity of the HPA axis that increases the risk of a suicide attempt [33].

Some reports state that people who have traumatic experiences in childhood have changes in the HHA axis, which is closely related to suicide. However, more evidence on the relationship between suicide and the HPA axis are needed to complete the knowledge about this fact. This would be a major breakthrough that would facilitate the understanding of the physiological alterations that occur in those who commit suicide or engage in suicidal behaviour.

#### Serotonin

Some remarkable findings have been found that would link suicidal behaviour to changes in serotonin levels. Some of these alterations affect its main metabolite, 5-hydroxyindoleacetic acid (5-HIAA), 5-HT receptors and the serotonin transporter (SERT) [6, 8, 11, 28, 44, 45, 47]. Some studies agree that reduced levels of 5-HIAA appear in those who attempt suicide. Low levels of 5-HIAA are also linked to the use of more lethal methods of suicide [47]. Likewise, decreased serotonin neurotransmission is related to impulsivity and aggression [31, 47]. In addition to reduced levels of serotonin transport [8]. However, other studies indicate that IL-1 β and TNF-α help to enhance serotonin transport, thus calling into question the claims of the other studies [7, 35].

#### Immune and infectious disorders

Another interesting finding about suicide is its relationship with autoimmune disorders such as multiple sclerosis or systemic lupus erythematosus. An increased risk of suicide has been found in patients with one of these disorders, as well as an increase in patients who develop major depression. It is not surprising that these disorders affecting the immune system are related to suicide. Therefore, those disorders that involve prolonged activation of the immune system will have an increased likelihood of developing depression or dying by suicide. Some studies that have investigated this fact suggest that 40% of patients with multiple sclerosis were also depressed and up to 15% of them eventually died by suicide. In addition, an increase in IL-12 has been found in these patients. Among those with haemophilia, 1% of them have made suicide attempts [7]. It has been also found that people with systemic lupus erythematosus are up to four times more likely to develop depression than those without the disorder. In addition, a high percentage of these people have psychiatric symptoms. However, it is currently unknown whether the increased risk of developing depression or dying by suicide is due to these psychiatric symptoms or to systemic lupus erythematosus [7]. In these disorders, interferon treatment may trigger a worsening of depressive symptoms or an increase in suicidal ideation. If we recall, neuroinflammation began to gain importance with the discovery of the link between both [7].

#### Other physiological alterations

In addition to the alterations mentioned above, the neural plasticity of people who die by suicide shows remarkable changes. There are studies that point to altered levels of brain-derived neurotrophic factor or BDNF. This may be an indicator of suicide risk. However, other studies state that there is no direct relationship between the two events, so more research is needed to know whether this would be an indicator to be considered [8, 47].

There are also contradictions about the influence of lipids on the risk of dying by suicide. Some studies claim that elevated levels of high-density lipoproteins (HDL), may trigger an increased risk of suicide. On the contrary, others indicate that the relationship is just the opposite or that there is no relationship between the two [2]. Another controversial finding is the increase in IL-6 related to the increase in body mass index. Some studies support this influence and others claim that there is no relationship between both [26].

Although more research is needed on the relationship between vitamin D levels and suicide, it is widely believed that lower levels may influence the risk of major depressive disorder and suicide. Vitamin D inhibits the synthesis of inflammatory markers, such as IL-6 or TNF-α, whereas decreased vitamin D levels coincides with increased levels of inflammatory markers [7].

## Conclusions

Suicide is one of the biggest health problems in our society, leading thousands of people to death every year. That is why it is essential to shed light on this phenomenon and claim its importance in institutions, scientific research and society. In order to reduce the silence and the social taboo that exists today, it is necessary to address suicide, to try to understand the extent of the problem and to investigate possible causes that lead a person to take his or her own life. That is why many people are already betting on a multidisciplinary approach to reach all aspects and details of suicide.

Suicide is considered a real challenge for mental health and, despite great scientific progress, more research is needed to understand the phenomenon and to develop effective prevention measures. Subjective ways of detecting suicide risk, such as questionnaires or scales, do not offer an optimal detection mechanism. However, it may be that in addition to objective means, such as studies on physiological alterations, favourable results can be achieved and a decrease in the risk of suicide may be achieved. The evidence tells us that current preventive measures do not succeed in curbing this phenomenon, which is why research in the field of neuroinflammation and other physiological alterations may be the solution. As stated by several authors, future directions include employing novel techniques to improve the prediction of suicidal behaviour, testing and applying theoretical models of suicidal behaviour, harnessing new technologies to monitor and intervene in suicide risk, expanding suicide prevention activities to low and middle-income countries, moving toward a more refined understanding of sub-groups of people at risk and developing tailored interventions. Also, there is a need for comparable information about attempted suicide across different countries and cultures.

In the twenty-first century, suicide and mental health are on the table of scientific research, but there is still a long way to go and more studies are needed to break the silence and to highlight their importance.

## Data Availability

No data was used for the research described in the article.
